# Lignosulfonates as Surfactants to Stabilize Elemental Sulfur Dispersions

**DOI:** 10.3390/polym17243288

**Published:** 2025-12-11

**Authors:** Tatiana N. Lugovitskaya, Denis A. Rogozhnikov

**Affiliations:** Institute of New Materials and Technologies, Ural Federal University named after the First President of Russia B.N. Yeltsin, 19 Mira St., Yekaterinburg 620002, Russia; darogozhnikov@urfu.ru

**Keywords:** lignosulfonates, elemental sulfur, stabilization, sol, vesicles, suspension, phase formation, coagulation

## Abstract

During sulfite delignification of wood, sulfo derivatives of lignin—lignosulfonates (LS)—are formed as a byproduct. Due to their amphiphilic nature, LS are used as plasticizers, dispersants, and stabilizers. The functions and performance characteristics of this surface-active polyelectrolyte are determined by its behavior in aqueous solution, at surfaces and interfaces, which, in turn, is determined by its chemical composition. This study investigated the effect of LS with various molecular weight compositions (M_w_ 9–50 kDa) on the behavior and aggregation stability of aqueous dispersions of elemental sulfur (S^0^) under conditions simulating hydrothermal leaching of sulfide ores. Using conductometry, potentiometry, tensiometry, and viscometry, a detailed study of the physicochemical properties of aqueous LS solutions (*C*_LS_ 0.02–1.28 g/dm^3^) obtained from a few sources (Krasnokamsk, Solikamsk, and Norwegian Pulp and Paper Mills) was conducted. The composition, molecular weight, and concentration of LS were found to significantly affect their specific electrical conductivity, pH, intrinsic viscosity, and surface activity. LS introduction during the formation of sulfur sols is shown to promote their stabilization through electrostatic and steric mechanisms. Optimum dispersion stability (293 K, pH 4.5–5.5) was observed at moderate LS concentrations (0.02–0.32 g/dm^3^), when a stable adsorption layer forms on the surface of sulfur particles. High-molecular-weight LS samples provided more effective spatial stabilization of sulfur particles. It has been established that increasing temperature (293–333 K) and changing pH (1–7) significantly affect the aggregative stability of systems; specifically, the sol stability decreases with increasing temperature, and the stabilizing effect of different LS types reverses upon changing pH. The obtained results highlight the potential of using naturally occurring polymeric dispersants to control the aggregation stability of sulfur-containing heterophase systems and can be applied to the design of stable colloidal systems in chemical engineering and hydrometallurgy.

## 1. Introduction

**Figure 1:**
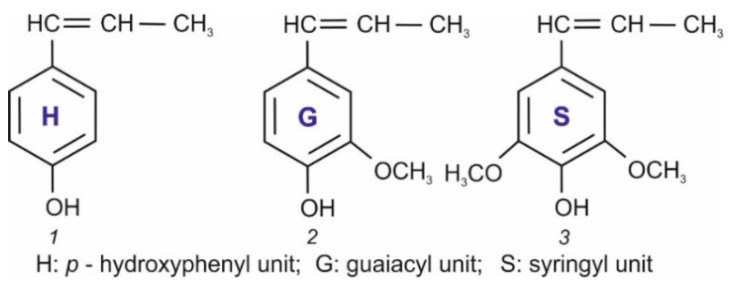
Monolignols, the precursor molecules responsible for the intrinsic lignin structure (adapted from Ref. [2]).

Lignin is a natural branched aromatic polymer that, along with cellulose and hemicelluloses, is a component of vascular plants, including woody plants, herbs, some algae, ferns, and horsetails [1]. Its structure has not yet been precisely determined. However, it is generally accepted that the lignin macromolecule is a combination of three phenylpropane monomers derived from certain precursor compounds known as monolignols, namely, *n*-coumaryl, coniferyl, and sinapic alcohols (Figure 1) [2].

Monolignols randomly link together to form a complex, branched, and irregular polymer network. This process is extremely complex and involves a series of radical and ionic reactions, resulting in the formation of a 3D lignin macromolecule [3].

Currently, it is impossible to isolate lignin in its original form. However, a number of its technical derivatives are available. For instance, sulfo derivatives of lignin (lignosulfonates, LS) are formed during sulfite delignification of wood as a byproduct. In this process, sulfonate groups are introduced into the polyaromatic backbone of lignin, and a few of the original lignin–lignin bonds are hydrolyzed [4]. The presence of a chaotic set of fragments and various functional groups determines the broad molecular weight distribution and polyfunctionality of LS samples [5]. The presence of hydrophobic (aromatic rings) and hydrophilic (sulfonate and carboxyl) groups prone to ionization in the macromolecular system of LS allows them to be considered as surface-active polyelectrolytes. The same properties underlie the use of LS as concrete modifiers, in oil well drilling fluids, colloid/suspension stabilizers, animal feed additives, etc. [6,7,8,9].

Compared to classical surfactants, LS exhibit low surface activity at the liquid–gas interface. According to literature data, the critical association concentration (CAC) varies over a wide range of LS concentrations and depends on many factors (sample purity, molecular weight, degree of sulfonation, pH, temperature, etc.). It has been shown [10] that the CAC in aqueous LS solutions obtained under laboratory conditions depends on the molecular weight and degree of sulfonation of the samples and varies within 16.72–40.06 g/dm^3^. Ge et al. [11] carried out deep preliminary cleaning and ultrasound treatment of technical LS samples. Under such conditions, the formation of micellar associates was observed at a concentration of 0.3 g/dm^3^. Li and Ouyang [12] estimated the CAC of narrow-dispersed LS fractions by fluorescence spectroscopy and found that with an increase in the molecular weight (4.7–26.7 kDa) of the samples, the CAC decreased from 0.24 to 0.15 g/dm^3^. The introduction of indifferent electrolytes [13], alcohols [14,15], and surfactants [16] into the binary “LS-water” system makes it possible to regulate the surface activity of LS.

Regarding the behavior of LS at the liquid–solid and liquid–liquid interfaces, they exhibit dispersing, emulsifying, and stabilizing effects with respect to a wide range of fillers (coalin, talc, carbon black, cement, oil, etc.) [17,18].

The hydrophilic–lipophilic balance (HLB) is a criterion for the efficiency of surfactant application (emulsifiers, stabilizers, or dispersants). The semi-empirical HLB system proposed by Griffin (1949) and developed by Davis (1957) is most often used as a first approximation. It allows for the quantitative evaluation, from an energetic perspective, of the degree of interaction between the functional groups of surfactant molecules and water or an organic phase, and is expressed as arbitrary group numbers [19,20]. It is generally accepted that an HLB in the range of 3–6 is typical for water-in-oil (W/O) emulsifiers, 6–8 for wetting agents, and 8–18 for oil-in-water (O/W) emulsifiers and solubilizers, respectively. Regarding LS, an approximate HLB of 11.6 was calculated [21,22] based on their elemental composition. Given the presence of sulfonate groups and polyaromatic structure, LS are assumed to have a greater affinity for aromatic oils than for paraffinic ones. Based on their behavior, LS are classified as stabilizers of oil-in-water emulsions with an effective HLB of 10–18 [23].

Several key mechanisms of the dispersing/emulsifying and stabilizing action of LS in heterogeneous systems have been revealed.

Sulfonate groups are ionized in an aqueous medium, and the LS macromolecule has a high negative charge density [24]. When LS adsorbs on the surface of solid particles/liquid droplets, a same-sign charge arises, which, at a sufficient ionic strength, causes repulsion between the particles, reducing the thickness of the electrical double layer (electrostatic stabilization) [25]. In addition, the adsorbed LS molecules are capable of creating a physical barrier around the particles, preventing their close contact (sticking/coalescence) and further facilitating dispersion/emulsification (steric stabilization). With an increase in the ionic strength of the medium, screening of the charges on the LS chain occurs, which may reduce electrostatic and enhance hydrophobic interactions between the phases. A change in the pH of the medium also leads to a change in the degree of dissociation of the hydrophilic LS groups, and, as a consequence, of the charge of the macromolecule and its conformation (unwinding/folding of chains, hydration). The latter is reflected in the ability of LS to adsorb and stabilize surfaces.

Temperature is another factor that significantly influences the interfacial processes in LS-containing suspensions/emulsions [26]. Temperature affects adsorption kinetics, macromolecular mobility, hydration processes, and all types of interactions (electrostatic, hydrophobic, etc.). An increase in temperature usually promotes aggregation and reduces the stability of suspensions.

The literature notes that LS adsorption on solid surfaces is often described by the Langmuir isotherm; there are also data on kinetic adsorption models (including pseudo-second-order ones) [27].

Thus, LS are highly effective dispersants/stabilizers/emulsifiers, representing a more environmentally friendly and cost-effective alternative to synthetic chemicals.

As surface-active polymers, LSs are widely used in the hydrometallurgical processing of ores [28]. For instance, in the autoclave leaching of sulfide concentrates according to Equation (1),

2MeS + 2H_2_SO_4_ +O_2_ → 2MeSO_4_ +2H_2_O + 2S^0^ (1)

in addition to the main products, elemental sulfur S^0^ is formed, which, at the process temperatures (≥393 K), forms a melt, resulting in sulfide dissolution ceasing [29,30]. LS addition eliminates the occluding effect of sulfur. However, over time and depending on the composition, the efficiency of LS decreases.

To control sulfide dissolution processes and achieve high recovery rates of valuable metals, it is necessary to elucidate the mechanism of LS action under conditions simulating autoclave/atmospheric leaching of ores. We comprehensively studied the interphase interactions in the LS–metal sulfide system [31]. LSs have been shown to exhibit an adsorption-wedging mechanism of action with respect to zinc sulfide. However, single studies [32,33] indicate that LS could also alter the nature of the elemental sulfur surface.

As a simple substance, elemental sulfur S^0^ is an amorphous yellow powder. It has a large number of various allotropic modifications due to the high ability of its atoms to combine with each other to form ring or chain molecules. The stable form of S^0^ is considered to be the orthorhombic allotropic modification α(β)-sulfur, consisting of cycloocta-S_8_ molecules [34,35]. At 368.3 K, α-sulfur becomes monoclinic, with a melting point of 392.6 °C. S^0^ is insoluble in water and has limited solubility in non-polar liquids at room temperature.

Thus, given that S^0^ is an inevitable product of the hydrochemical processing of sulfide concentrates and, due to its physicochemical properties (tendency to melt and aggregate), it is capable of significantly influencing the process, studying its behavior in the presence of LS is of particular interest [36]. Understanding the characteristics of the interaction of LS with dispersed sulfur is necessary to establish the mechanisms for stabilizing the resulting sulfur particles and, consequently, to improve the efficiency of hydrometallurgical processes.

The aim of this work was to establish the relationship between the structural and molecular characteristics of LS and their influence on the physicochemical properties of aqueous solutions, as well as to reveal patterns of change in the aggregative stability of elemental sulfur sols in the presence of LS from the moment of their formation until the loss of aggregative and sedimentation stability under conditions simulating the temperature and pH of the hydrothermal treatment of ores.

## 2. Materials and Methods

### 2.1. Materials

**Figure 2:**
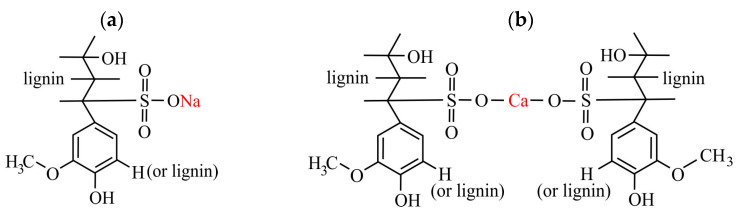
Proposed structures of the phenylpropane unit of the LS studied: (**a**)-LS1, LS2, (**b**)-LS3.

The following substances were used: a technical sample of LS from the Krasnokamsk Pulp and Paper Mill (LS1) (KAMA Ltd., Perm City, Russia) and LS fractions with different molecular weights, isolated by preparative ultrafiltration (1.5 MPa, 303–308 K) from industrial sulfite liquors of the Solikamsk (LS2) (Solikamskbumprom Corp., Solikamsk town, Russia) and Norwegian (LS3) (Borregaard Corp., Sarpsborg, Norway) pulp and paper mills. The proposed chemical structures of the LS used (in a simplified form) are shown in Figure 2.

The mass-average molecular weights (${\bar{M}}_{w}$) of the LS, estimated by the sedimentation equilibrium method, were 18.6, 9.25, and 46.3 kDa for LS1, LS2, and LS3, respectively. Table 1 presents the elemental and functional compositions of the LS used.

Finely dispersed powder of elemental sulfur S^0^, special purity grade 15-3 (Lenreaktiv Corp., St. Petersburg, Russia); ethyl alcohol EtOH, sulfuric acid H_2_SO_4_, sodium hydroxide NaOH (chemically pure grade) were used without further purification; distilled water was also utilized.

### 2.2. Study Objects

The objects of our study were aqueous LS solutions and LS-stabilized elemental sulfur sols. Aqueous solutions with LS concentrations (*C*_LS_) of 0.10–1.28 g/dm^3^ were obtained by dissolving a weighed portion of the polymer in a calculated volume of water at a temperature of 293 ± 2 K and stirring with a magnetic stirrer until the LS was completely dissolved. The prepared LS solutions were dedusted using a Millipore filter with pore diameters of ≤0.45 μm.

To prepare S^0^ sols, elemental sulfur powder was first dissolved in EtOH (0.2 g/dm^3^). Then, an aliquot (2–5 mL) of this S^0^ solution was added dropwise to the aqueous LS solution of constant volume (10 mL) and concentration (*C*_LS_ 0.002–0.640 g/dm^3^). The mixture was shaken for one minute, and then the change in transmittance (*T*, %) at a wavelength of λ 440 nm was recorded over time (τ, 0–1440 min). The *T* of the sol was measured from the moment of S^0^ nucleation and the formation of equilibrium (stabilized) macrodisperse structures until the loss of aggregation and sedimentation stability (complete clarification of the sol) of the S^0^ dispersions. Based on the obtained data, kinetic dependences of suspension clarification *T* = *f*(τ) were plotted, and their lifetime (τ_s_) and degradation rate *V*_k_ (ascending sections on *T* = *f*(τ)) were estimated.

A series of experiments determined the effect of low-molecular-weight electrolyte additives (NaOH and H_2_SO_4_) and temperature (293–333 K) on the aggregative stability of S^0^ sols.

### 2.3. Methods

**Elemental analysis** was performed on a VarioMICROcube analyzer (Elementar, Langenselbold, Germany). The accuracy was ±0.5 wt.%.

Barium titration was used to analyze sulfur content. The accuracy ranged within 5–10%. Methoxyl group content was evaluated using conventional hydroiodine treatment and gas chromatography [37].

**Hydrogen index (pH)** was measured using a Mettler Toledo Five Easy FE20 pH meter (MTD, Singapore). The system was calibrated using NIST Traceable calibration buffers with pH 4.01, 7.00, and 10.01.

**Electrical conductivity** was evaluated by measuring resistance on a WTW inoLab Cond 7110 conductivity meter (WTW, Troistedt, Germany) with an accuracy of ±0.5%. A thermostatted 25 mL cell was used. Specific electrical conductivity (æ_sp_, S/cm) was calculated based on data from three replicate measurements and taking into account the specific conductivity of water using Equation (2):

æ_sp_ = *K*/*R*-æ_H2O_, (2)

where *K* is the cell constant, cm^−1^; *R* the solution resistance, S; and æ_H2O_ is the specific electrical conductivity of water, S/cm.

**Gravimetric measurements** were performed on an Ohaus Discovery analytical balance (USA), with an accuracy of ±0.0001 g.

**The transmittance** (*T*, %) of sols was recorded on an Analytik Jena spectrophotometer at a wavelength of 440 nm.

**Surface tension (σ_l–g_, J/m^2^)** was measured using the Wilhelmy method.

**Viscometric studies** were performed on a Ubbelohde viscometer with a capillary diameter of 0.54 mm within a temperature range of 293–353 K. The thermostatting time was 15–20 min. The flow times of the polymer solution (*t*, s) and the solvent mixture (*t*_0_, s) were measured with an accuracy of ±0.1 s, and the viscosity index (η_sp_/*C*_LS_, dL/g) was calculated by Equation (3):

(3) $$\frac{\eta_{sp}}{C_{LS}}=\frac{\left.t-t_{0}\right.}{t_{0}}\cdot C_{LS},$$

where *C*_LS_ is the LS concentration, g/dL. The intrinsic viscosity ([η], dL/g) was calculated using the Huggins Equation (4).

(4) $$\eta_{sp}=\left[\eta \right]\cdot C_{LS}+k_{H}\cdot {\left(\left[\eta \right]\cdot C_{LS}\right)}^{2},$$

where *k*_H_ is the Huggins constant.

The **morphology** of S^0^ crystals was assessed using a MIRA II LMU scanning electron microscope (SEM). A layer of colloidal gold approximately 70 Å in thickness was pre-sputtered onto the sample using an Emitech K4 setup.

## 3. Results and Discussion

Before discussing the behavior of LS in heterogeneous systems based on elemental sulfur, let us examine the properties of aqueous LS solutions.

### 3.1. LS Behavior in an Aqueous Medium

When dissolved in water, LSs form colloidal solutions with a typical Tyndall effect [38]. Their physicochemical constants are presented in Table 2.

Analysis of the data (Table 1 and Table 2) reveals differences between the LS samples studied in terms of elemental composition, the presence of functional groups, and molecular weight. In particular, LS3 has a higher molecular weight and a lower sulfonation degree compared to LS1 and LS2. This explains the differences in the nature of changes in the physicochemical properties of their aqueous solutions.

When dissolved in water, LSs are ionized. Taking into account the p*K* values of sulfonate, carboxyl, and phenolic hydroxyl groups, which are 1.5, 5.1, and 10.5, respectively [40], sulfonate groups are ionized to the greatest extent in the studied concentration range (0.20–1.28 g/dm^3^). Dissociation processes are accompanied by changes in the specific electrical conductivity of LS solutions (Table 2). With an increase in the concentration of *C*_LS_ and a decrease in the molecular weight of the samples, an increase in the specific electrical conductivity (æ_sp_) was observed. However, this increase was not linear, caused not only by ionization processes, but also by partial counterion condensation on the LS polyanion. For instance, in the concentration range of ~0.2–0.5 g/dm^3^, the LS macromolecule dissociates to form a polyanion and free counterions, which corresponds to the polyelectrolyte regime. With a further increase in concentration (*C*_LS_ > 0.5 g/dm^3^), the total number of counterions increases, some of which remain free, while others bind to the polyanion to form ion pairs. This indicates a transition to a mixed polyelectrolyte–ionomer regime, accompanied by a slower rate of increase in æ_sp_. A similar effect of polyelectrolyte concentration on the electrical conductivity of solutions was previously established for other polymer systems [41,42,43].

Comparison of absolute conductivity values showed that the degree of ionization of LS1 and LS2 in an aqueous medium was higher than that of LS3. This may be due to some behavioral characteristics of polyanions in solution. With an increase in the number of hydrophobic substituents and the macromolecular size, the polyanion mobility decreases. Furthermore, long LS macromolecules (large organic ions) can participate in hydrophobic hydration, thereby slowing the translational movement of water molecules in solution [44].

The change in the pH of solutions with varying *C*_LS_ cannot be unambiguously interpreted, as it is determined by the LS type. For LS3 solutions, an increase in pH from 4.6 to 5.6 was observed with increasing concentration. For LS1 and LS2, with lower molecular weights, a concentration range of 0.15–0.17 g/dm^3^ was revealed, within which the maximum pH was observed, reaching 5.0–5.1 and 6.0–6.05, respectively. With a further increase in *C*_LS_ (above 0.16 g/dm^3^), a decrease in the pH was observed. This pH-concentration dependence is likely due to the parallel hydrolysis process characteristic of low-molecular-weight LS [45].

Despite their polymeric nature, LS are characterized by very low intrinsic viscosity [η], especially compared to synthetic polymers of similar molecular weights [39]. For example, the limiting viscosity number [η] was 0.015 and 0.028 dL/g for LS2 and LS3, respectively.

The main causes of this hydrodynamic behavior of LS macromolecules are their high structural branching, lack of macromolecular flexibility, and tendency to associate [46]. Such macromolecules, due to their amphiphilicity, are compactly coiled in solution, therefore occupying a small hydrodynamic volume despite their high molecular weight. Therefore, the hydrophobic regions of every macromolecule are shielded and interact with water to a very limited extent. Charges are also shielded (as evidenced by the change in specific conductivity), and the macromolecules are only partially stretched, unlike classic polyelectrolytes. The latter also reduces the hydrodynamic volume and, consequently, the intrinsic viscosity of aqueous LS solutions. Thus, low [η] values indicate that the LS macromolecules in aqueous solution swell to a significantly lesser extent than linear flexible-chain polymers, and they exist as compact particles exhibiting no asymmetry.

Depending on the manufacturer and the degree of LS purification, the crossover points for this polymer ranged from 29 to 47 g/dL.

Thus, studying the physicochemical properties of LS solutions allows us to reveal the key characteristics determining their behavior in an aqueous medium. Significant differences in the composition and properties of the LS samples depending on the manufacturer (Solikamsk, Krasnokamsk, and Norwegian Pulp and Paper Mills) were revealed as well.

The next stage of our study will be devoted to the investigation of sols obtained by mixing an LS solution with an alcohol solution of elemental sulfur. The resulting LS–S^0^–H_2_O–EtOH systems are heterogeneous dispersions. Studying their aggregation stability and lifetime, as well as the influence of various factors on their stability, is of significant scientific and practical interest and is necessary for understanding the mechanism of action of LS in the leaching of sulfide ores.

### 3.2. Effect of LS of Various Molecular Weight Compositions on the Aggregation Stability of Sulfur Sols

#### 3.2.1. Selecting Optimal Parameters for the Formation and Destruction (Coagulation) of S^0^ Sols

**Figure 3:**
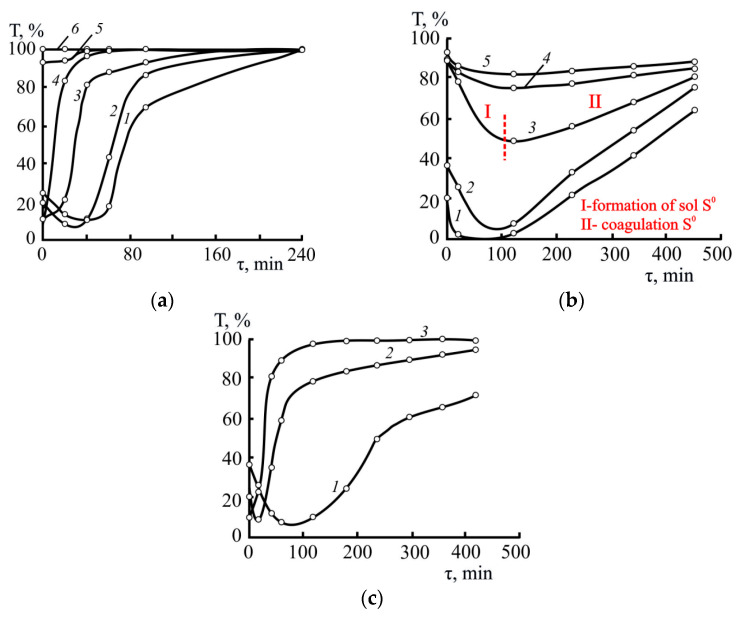
Kinetic dependences of the clarification of aqueous sulfur suspensions depending on the alcohol content (**a**), elemental sulfur content (**b**), and temperature (**c**). (**a**): 333 K; *C*_S_ = 0.04 g/dm^3^; EtOH, %: 1–4, 2–8, 3–16, 4–32, 5–46, 6–60. (**b**): 293 K; EtOH = 12%; *C*_S_, g/dm^3^: 1–0.08, 2–0.04, 3–0.02, 4–0.017, 5–0.014. (**c**): *C*_S_ = 0.03 g/dm^3^; EtOH = 10%; *T*, K: 1–293, 2–313, 3–333.

In a preliminary series of experiments, the sol composition was optimized in terms of alcohol and sulfur content by varying the volume of the sulfur-containing alcohol solution (Figure 3a–c). As an optimality criterion, ensuring better resolution during measurements, we took the range of changes in the optical transmittance coefficients (Δ*T* = 85–90%) of the sols from their formation until complete decomposition.

Increasing the alcohol content in sulfur-isoconcentrated sols was accompanied by a decrease in ΔT values due to the increased physical solubility of sulfur (Figure 3a). However, the suspension clarification rates, calculated for the same optical transmittance values, remained virtually unchanged.

Increasing the sulfur content in the sols had virtually no effect on the onset of their decomposition (minima in Figure 3b) or their overall lifetime (maxima in Figure 3b). However, it expanded the range of ΔT values and intensified the clarification processes (constrained coagulation regime). The descending section (for example, Figure 3b, region I) of the kinetic curves corresponds to the region of sol formation (formation and diffusion of solid-phase sulfur in the bulk), while the ascending section (Figure 3b, region II) corresponds to their aggregative instability associated with the development of coagulation processes.

Acceptable Δ*T* values (at least 80%) were observed in the sols with a sulfur content of 0.04 g/dm^3^; all subsequent experiments were conducted with suspensions of this composition.

#### 3.2.2. Effect of the Concentration and Type of LS

**Figure 4:**
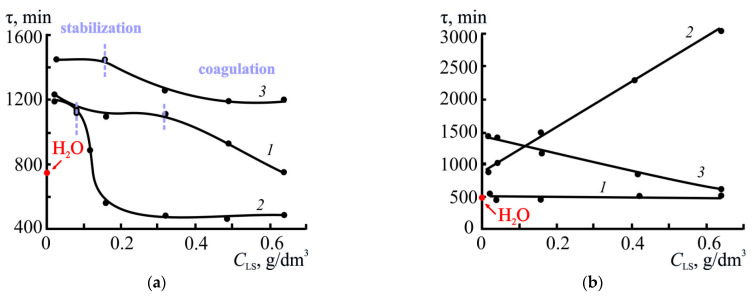
Effect of lignosulfonate concentration on the breakdown period of elemental sulfur suspensions at several pH values: (**a**)-4.5, (**b**)-1.0. 293 K; *T* = 80%; 1-LS1, 2-LS2, 3-LS3.

As can be seen from Figure 4a, the concentration and molecular weight of LS have the main influence on the stability of the S^0^ sols.

The presented τ = *f* (*C*_LS_) dependences indicated that the sol lifetime in the presence of LS1 and LS3 was higher than in the absence of LS across the entire range of studied concentrations. Two characteristic regions (highlighted by the dashed lines in Figure 4a) of change in the aggregation stability of the sols can also be identified in all dependences. For instance, the maximum system stability for LS1 was achieved in the concentration range of 0.02–0.32 g/dm^3^, while the sol lifetime was 1200–1210 min. With a further increase in *C*_LS1_ up to 0.64 g/dm^3^, a decrease in stability to 800 min was observed.

A similar pattern was observed for LS3: in the *C*_LS3_ range of 0.04–0.32 g/dm^3^, the sol lifetime was 1350–1450 min, after which τ decreased to 1200 min. The higher stability of the system in the presence of LS3 indicates a significant role of the molecular weight of the sample; specifically, more branched macromolecules likely provide more effective spatial stabilization of sulfur particles due to the formation of a strong adsorption layer and pronounced steric effects.

In the case of LS2, sol stability increased only within a narrow concentration range of 0.02–0.04 g/dm^3^ (1100–1200 min), after which τ dropped sharply and reached 500 min. This may indicate the unstable nature of the LS2 adsorption layer.

For comparison, in the absence of a stabilizer (in water), the sol’s lifetime was 790 min, which was significantly lower than the values obtained with the addition of LS samples. This confirms that all the LS used exhibited stabilizing activity, but the efficiency of this stabilization depended on both the molecular weight and concentration of LS.

Thus, the optimal stabilization of sulfur sols is achieved at moderate LS concentrations, when surfactant molecules form a stable adsorption layer on the surface of sulfur particles, preventing their coagulation. At higher concentrations, the formation of excess layers and subsequent destabilization of the system are likely. The sample with the highest molecular weight, LS3, had high efficiency, which indicates the predominant role of the steric stabilization mechanism.

When discussing the possible mechanisms of stabilizing sulfur sols in the presence of LS in more detail, it is important to keep in mind that during the formation of a water–alcohol sulfur sol, interactions between S^0^ particles occur under the influence of van der Waals forces [34,47]. In addition, solvation of sulfur particles by polar solvent molecules and electrostatic interactions (especially in aqueous solutions) are observed. The balance of these interactions, depending on the mixed solvent polarity, determines the stability and properties of the resulting sol.

During the formation of a sulfur sol in the presence of LS, adsorption of macromolecules on the surface of S^0^ dispersions occurs. Since LSs in solution are ionized, although the presence of EtOH will somewhat suppress dissociation, the adsorbed LS layer will impart a negative charge to the particle surface, thereby increasing the electrostatic repulsion between them. The latter prevents the aggregation/coagulation of sulfur particles. A similar stabilization mechanism was described for carbon dispersions and other solid particles in the presence of LS [17].

In our opinion, in addition to the electrostatic effect, steric stabilization of LS sulfur dispersions is possible and preferable. LS molecules (especially those with higher molecular weights) are capable of forming an adsorption/polymer layer around solid dispersions, preventing sulfur particles from approaching distances at which aggregation begins.

**Figure 5:**
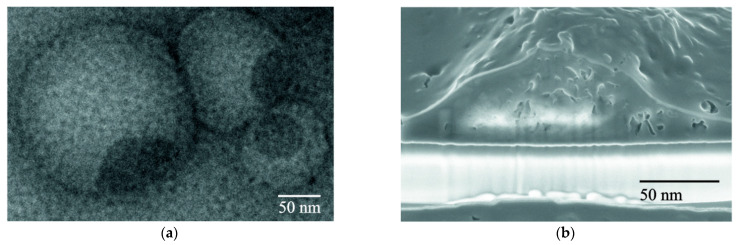
TEM (**a**) and SEM (**b**) images of lignosulfonate-containing associates isolated from water–organic media: *C*_LS3_ 0.8 g/dm^3^.

In a number of previous studies [48,49,50], we demonstrated the formation of vesicular LS structures in mixed water–organic media. Figure 5a,b show the general appearance of spontaneously formed LS associates.

Considering that S^0^ dispersions are formed in a mixed solvent of EtOH and H_2_O, the spontaneous formation of LS associates with the capture of sulfur particles, resulting in the creation of steric constraints (barriers) preventing their aggregation, is quite probable. It should also be noted that the LS samples with molecular weights greater than 40 kDa are prone to the formation of vesicular associates, which is also consistent with their (LS3) higher stabilizing activity.

**Figure 6:**
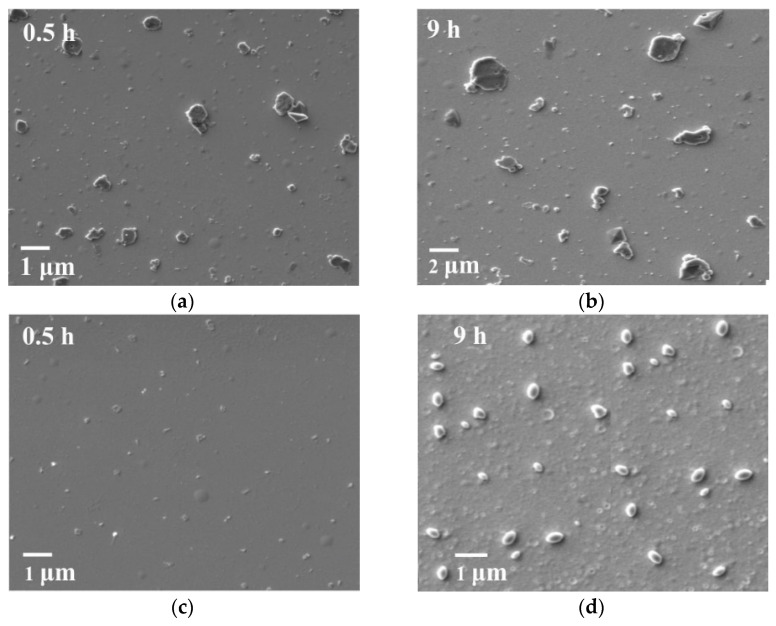
SEM images of S^0^ crystals formed in the absence (**a**,**b**) and in the presence of LS (**c**,**d**) over a 9 h sol lifetime.

It is also useful to track the change in the morphology of S^0^ crystallites over time from the moment of their formation. Figure 6 shows SEM images of S^0^ dispersions formed over time in the absence (Figure 6a,b) and in the presence of LS (Figure 6c,d).

The resulting images demonstrate a clear dependence of the size and morphological characteristics of S^0^ particles on the composition and lifetime of the sol. In the absence of LS, the formation of flat, lamellar crystalline structures of elemental sulfur was observed, the sizes of which increased over time: from 500–800 nm with a sol lifetime of 1 h up to 1.5–2.0 µm after 9 h of sol storage (Figure 6a,b).

In the presence of LS, bulk S^0^ crystals with an oval morphology were formed, the sizes of which also changed during the aging of the system (Figure 6c,d), ranging from 50 to 400 nm after 9 h of sol storage, which is 5–6 times smaller than the sizes of crystals formed in the absence of LS.

Thus, it can be concluded that the stabilization of sulfur sols under the influence of LS occurs through electrostatic and steric stabilization.

Next, the influence of various parameters (temperature, pH, and addition of electrolytes) on the stabilizing effect of LS will be discussed.

#### 3.2.3. Effect of Temperature

**Figure 7:**
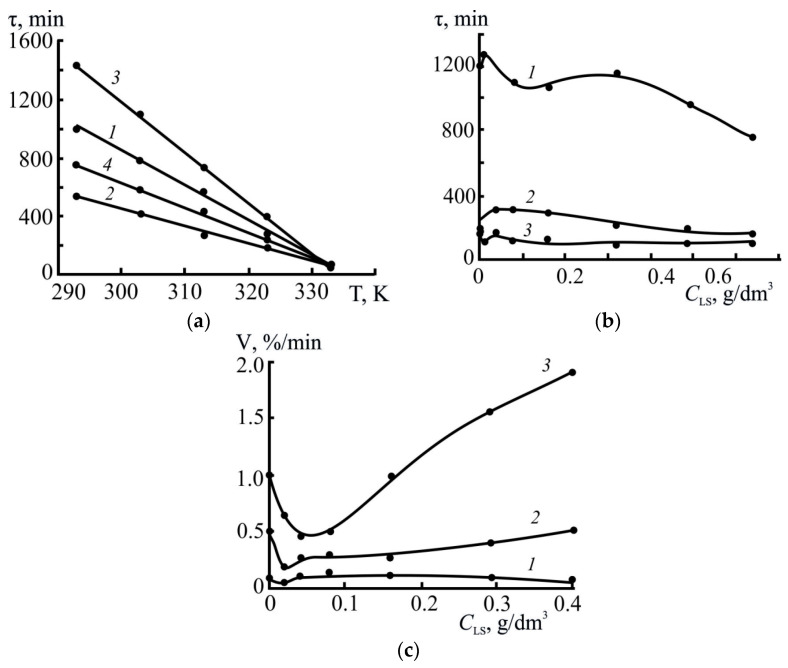
Effect of temperature (**a**) and LS concentration (**b**,**c**) on the lifetime and destruction rate of elemental sulfur suspensions (*T* = 80%). (**a**): *C*_LS_ = 0.16 g/dm^3^; *C*_S_ = 0.04 g/dm^3^; 1-LS1, 2-LS2, 3-LS3, 4-H_2_O. (**b**,**c**): LS1; *T*, K: 1–293, 2–313, 3–333.

As the temperature increased, the aggregative stability of the sols deteriorated (Figure 7a); with an increase in temperature from 293 to 313 K, the overall lifetime of the suspensions was reduced by 2 times in the absence of LS. In the same series, sulfur coagulation processes intensified; for instance, the rates of sol destruction at temperatures of 293, 313, and 333 K, calculated from the ascending portion of the kinetic dependences (in the range of *T* = 10–40%) were 0.2, 0.8, and 1.6%/min, respectively. The destabilizing effect of the temperature factor on sulfur sols is also noted in Ref. [51].

The destabilizing contribution of the temperature factor is equally evident in lignosulfonate-containing sols (Figure 7a–c). The sols containing LS2 had the shortest lifetime under all temperature conditions; moreover, the destabilizing effect increased (by at least 2 times) at higher concentrations of this LS (above 0.16–0.20 g/dm^3^). At low temperatures in isoconcentration (based on LS content) conditions, suspensions containing the high-molecular-weight sample of LS3 exhibited greater aggregation stability. The differences in the stability of suspensions containing LS of different compositions observed at low temperatures were leveled out as the temperature increased further (Figure 7a); for suspensions with equal contents of the three types of LS, the τ_s_ values at 333 K were almost the same (τ_s_ = 60–100 min).

In certain areas limited by the LS concentration (0.01–0.05 g/dm^3^), extreme changes in the coagulation rates of sulfur dispersions are observed (Figure 7c). As the suspension temperature decreased, the minima *V*_k_ shifted towards lower LS concentrations. For example, at 293 K, the minimum coagulation rate was observed at *C*_LS_ = 0.01 g/dm^3^ (Figure 7c). Beyond these concentration thresholds, the systems were less stable.

Thus, increasing temperature enhances thermal motion, reduces the viscosity of the medium, weakens the adsorption of LS macromolecules, and could also lead to LS desorption from the particle surface or the destruction of LS associates. Additionally, the differences between the LS samples are leveled out (at 333 K, the τ_s_ values for all three are almost identical). This suggests that stabilizing mechanisms become less significant at high temperatures, and thermal (entropic) aggregation processes dominate. It should also be noted that an increase in the growth rate of elemental sulfur dispersions with increasing temperature was also observed in the presence of other surfactants (poly(oxyethylene) p-tert-octylphenyl ether TX-100, sodium dodecylbenzenesulfonate, and cetyltrimethylammonium bromide, CTAB) [52].

#### 3.2.4. Effect of pH

**Figure 8:**
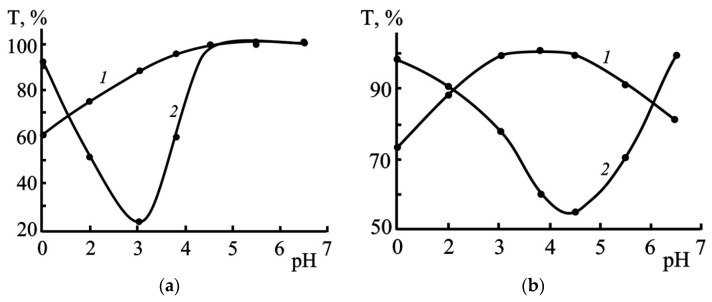
Effect of pH on the stability of elemental sulfur suspensions at temperatures of 293 (**a**) and 333 K (**b**). τ_S_: (**a**)-1440 min; (**b**)180 min; 1-*C*_LS1_ = 0.16 g/dm^3^; 2-*C*_LS_ = 0 g/dm^3^.

The aggregation stability of sols depending on the pH of the medium (1.0–6.5) was studied by varying the quantitative contents of sulfuric acid and sodium hydroxide. In the LS-free sols, extreme changes in aggregation stability were observed at all temperatures, depending on pH. The maximum stability of the suspensions (minima *T* in Figure 8) at temperatures of 293 and 333 K corresponds to the pH ranges of 2.5–3.5 and 4.0–5.0, respectively. Aggregation stability deteriorates beyond these pH ranges.

Under comparative conditions of temperature and LS concentration, pH ranges are revealed in Figure 8, where the sols exhibit clear differences in stability. The LS-free sols, compared to those containing lignosulfonates, were less stable when pH < 1.8–2.0, while the opposite trend was observed within the range 2.0 < pH < 4.5. At elevated temperatures (333 K), the LS-containing systems also stabilized in the pH range above 5.0 (Figure 8).

The influence of pH and the concentration of various types of LS on the lifetime of sols is also reflected in the dependences presented in Figure 4a,b. Their analysis indicates that in the media with no electrolytes (pH 4.5), the sols containing the high-molecular-weight sample of LS3 exhibited the greatest aggregation stability; the lowest stability was observed in suspensions with LS2 (τ_s_ = 550–600 min when *C*_LS_ > 0.16 g/dm^3^).

With a further increase in acidity (pH 1), an inversion in the stabilizing capacity of LS was observed; specifically, sols with LS2 were characterized by greater stability. Moreover, the effect of LS activity reversal at pH = 1 intensified as their concentration increased., e.g., at insignificant contents (0.08–0.10 g/dm^3^) of three LS types (LS1, LS2 and LS3) in suspensions their lifetime at pH 1 was 450, 1000 and 1200 min, respectively; with a 5-fold increase in the LS concentration (*C*_LS_ = 0.4–0.5 g/dm^3^), the τ_s_ values in the suspensions with LS2 increased by at least 2 times (2100–2500 min), and in the presence of the other two types, they remained virtually unchanged.

The differences in the stability of sols, determined (at comparable pH values) by compositional features of LS, are undoubtedly due to both the electrostatic contribution of ionic forms (sulfuric acid) to the development of interfacial processes involving sulfur dispersions and the physicochemical transformations of the LS themselves, in particular their ability to form associates [53]. For instance, upon the introduction of a low-molecular-weight electrolyte into the S^0^–LS sol, the degree of dissociation of the LS sulfonate groups will be significantly reduced, and the association processes of the LS will be intensified. In this case, steric factors in sol stabilization will predominate. The size of the resulting LS associates will likely also be important. As shown by Mingfang Yan et al. [54], the size of LS associates in aqueous solutions naturally decreases with a decrease in pH and, as a consequence, their surface-active properties change.

Forced pH changes also indirectly affect the spontaneous hydration processes of LS.

Thus, lignosulfonates of various types exhibit effective stabilizing properties with respect to elemental sulfur dispersions, ensuring their long-term aggregative stability. The most pronounced effect at pH 4.5–5.5 is observed for high-molecular-weight LS samples, acting primarily via a steric mechanism; in the more acidic range (pH 1), sols in the presence of LS2 are stable. The obtained results are of practical importance for understanding the mechanism of action of LS in hydrothermal and autoclave processing of sulfide ores, where the formation and stabilization of elemental sulfur significantly affect the kinetics of leaching of valuable metals.

## 4. Conclusions

Lignosulfonates are amphiphilic polyelectrolytes capable of forming stable colloidal solutions and associates in aqueous systems. Their physicochemical properties (specific conductivity, pH, intrinsic viscosity, and surface tension) depend on their elemental and functional composition, molecular weight, and concentration of the samples in solution.

The introduction of lignosulfonates during the formation of elemental sulfur sols has a stabilizing effect; specifically, all studied lignosulfonates increased the aggregative stability of sulfur sols, extending their lifetime compared to pure aqueous dispersions. However, the efficiency of stabilization is determined by the type and concentration of lignosulfonates, and external conditions (temperature, pH). The optimal lignosulfonate concentrations at 298 K are 0.02–0.32 g/dm^3^, which ensures the formation of a stable adsorption layer on the surface of sulfur particles. Above this range, the stability of sols decreases.

The molecular weight of LS has a decisive influence on the stabilization mechanism. The high-molecular-weight sample (LS3, $\bar{M_{w}}$ = 46.3 kDa) provides the greatest sol stability due to pronounced steric stabilization, while low-molecular-weight LS, due to their greater ionization, act primarily through electrostatic mechanisms.

Morphological analysis (SEM) of elemental sulfur dispersions showed that the S^0^ particles were smaller (50–400 nm) and more rounded in the presence of LS, whereas without the addition of LS, large plate-like crystals (up to 2 μm) formed. This confirms the stabilizing effect of LS.

Increasing temperature (293–333 K) accelerated coagulation and reduced the stability of S^0^ sols, which may be due to desorption of LS macromolecules and increased Brownian motion in the dispersions.

pH also significantly influences the stability of systems. The maximum stability of sols without LS was observed within pH ≈ 2.5–3.5, while the range widened in the presence of LS, especially at low pH values, where LS2 proved to be the most active stabilizer.

Our studies of the stabilizing effect of lignosulfonates of various compositions will expand our understanding of the behavior of amphiphilic natural polymers in hydrothermal systems and will also enable researchers to select the optimal LS composition and dosage for processing different types of ores, regulate the acidity of the medium and temperature regime, thereby preventing secondary sulfur precipitation.

Further research will focus on finding ways to increase the stabilizing effect of lignosulfonates with respect to S^0^ by developing surface-active compositions LS–surfactants from the classes of anionic (sodium dodecylsulfate), cationic (cetyltrimethylammonium bromide), and nonionic (Tween 80) surfactants.

## Figures and Tables

**Table 1:** Characterization of the lignosulfonate samples used.

Sample	Content, wt.%								${\bar{M}}_{w}$, kDa
	C	H	O	S	Me^n+^	SO_3_H	OCH_3_	OH_phen_	
LS1	33.9	4.72	46.8	9.5	Na 5.7	13.4	11.3	2.56	18.60
LS2	38.82	4.36	42.35	5.50	Na 6.6	12.68	10.6	2.32	9.25
LS3	48.70	4.52	38.20	4.24	Ca 3.0	12.30	9.2	2.10	46.30

**Table 2:** Physicochemical properties of aqueous LS solutions.

*C*_LS_, g/dm^3^		Indices		
		pH	æ_sp_ × 10^−5^_,_ S/m	σ_l–g_ × 10^−3^, J/m^2^
LS1	0.01	4.50	0.51	72
	0.02	4.60	0.90	72
	0.04	5.10	1.10	81
	0.08	5.00	1.40	78
	0.16	4.90	2.75	72
	0.32	4.90	4.45	65
	0.64	5.20	8.20	62
LS2	0.01	4.85	0.42	66
	0.02	5.13	0.78	64
	0.04	5.46	1.57	72
	0.08	5.71	3.01	68
	0.16	6.05	5.36	70
	0.32	5.80	10.46	70
	0.64	5.34	18.17	70
	Intrinsic viscosity [η]-0.015 dL/g;			
	Crossover-47 g/dL [39].			
-	0.02	4.60	0.25	72.25
	0.04	4.80	0.29	70.12
	0.08	4.70	0.54	70.12
	0.16	5.30	0.92	69.10
	0.32	5.25	1.47	69.10
	0.64	5.50	3.14	68.24
	1.28	5.56	4.23	68.24
	Intrinsic viscosity [η]-0.028 dL/g;			
	Crossover-29 g/dL [39].			

## Data Availability

The original contributions presented in this study are included in the article. Further inquiries can be directed to the corresponding author.

## Abbreviations

The following abbreviations are used in this manuscript:

CAC	critical association concentration
HLB	hydrophilic-lipophilic balance
LS	lignosulfonates
O/W	oil-in-water
S°	elemental sulfur
SEM	scanning electron microscope
TEM	transparent electron microscope
W/O	water-in-oil
